# Iron homeostasis and oxidative stress in idiopathic pulmonary alveolar proteinosis: a case-control study

**DOI:** 10.1186/1465-9921-9-10

**Published:** 2008-01-23

**Authors:** Andrew J Ghio, Jacqueline G Stonehuerner, Judy H Richards, Kay M Crissman, Victor L Roggli, Claude A Piantadosi, Martha Sue Carraway

**Affiliations:** 1 National Health and Environmental Effects Research Laboratory, Environmental Protection Agency, Research Triangle Park, NC 27711, USA; 2 Departments of Pathology and Medicine, Duke University Medical Center, Durham, NC 27710, USA; 3 Department of Medicine, Duke University Medical Center, Durham, NC 27710, USA

## Abstract

**Background:**

Lung injury caused by both inhaled dusts and infectious agents depends on increased availability of iron and metal-catalyzed oxidative stress. Because inhaled particles, such as silica, and certain infections can cause secondary pulmonary alveolar proteinosis (PAP), we tested the hypothesis that idiopathic PAP is associated with an altered iron homeostasis in the human lung.

**Methods:**

Healthy volunteers (n = 20) and patients with idiopathic PAP (n = 20) underwent bronchoalveolar lavage and measurements were made of total protein, iron, tranferrin, transferrin receptor, lactoferrin, and ferritin. Histochemical staining for iron and ferritin was done in the cell pellets from control subjects and PAP patients, and in lung specimens of patients without cardiopulmonary disease and with PAP. Lavage concentrations of urate, glutathione, and ascorbate were also measured as indices of oxidative stress.

**Results:**

Lavage concentrations of iron, transferrin, transferrin receptor, lactoferrin, and ferritin were significantly elevated in PAP patients relative to healthy volunteers. The cells of PAP patients had accumulated significant iron and ferritin, as well as considerable amounts of extracellular ferritin. Immunohistochemistry for ferritin in lung tissue revealed comparable amounts of this metal-storage protein in the lower respiratory tract of PAP patients both intracellularly and extracellularly. Lavage concentrations of ascorbate, glutathione, and urate were significantly lower in the lavage fluid of the PAP patients.

**Conclusion:**

Iron homeostasis is altered in the lungs of patients with idiopathic PAP, as large amounts of catalytically-active iron and low molecular weight anti-oxidant depletion are present. These findings suggest a metal-catalyzed oxidative stress in the maintenance of this disease.

## Background

Pulmonary alveolar proteinosis (PAP; also alveolar proteinosis and alveolar lipoproteinosis) has been classified as a disease entity since 1958 [1]. PAP is characterized by the massive accumulation of surfactant in the airspaces of the lower respiratory tract [2,3]. Pulmonary surfactant is a mixture of phospholipids and proteins essential for lung homeostasis and alveolar stability. The accumulation of surfactant in alveolar proteinosis appears to result from impaired phospholipid clearance rather than over-production by type II pneumocytes [4]. PAP is also characterized by inflammation with accumulation of abnormal alveolar macrophages containing intracellular surfactant-like material, and these phagocytes demonstrate impaired function [5,6]. Hyperplasia of type II pneumocytes, infiltration of lymphocytes and fibroblasts, and fibrosis can also be observed in PAP [7-9].

In most cases, PAP is idiopathic or primary, but secondary PAP can accompany inhalational dust exposures (e.g. silica), infections (especially *Mycobacteria*, *pneumocystis carinii*, *nocardia*, and *cryptococci*), malignancies, and immunosuppressive disorders [10,11]. The etiological factors in primary PAP remain unknown, but the development of antibodies to GM-CSF has led to the postulate that it is an auto-immune disorder [2]. Lung damage from infectious agents and inhaled dusts is promoted by the availability of iron and metal-catalyzed oxidative stress [12,13]. We tested the hypothesis that primary PAP can be associated with altered iron homeostasis in the lung by measuring concentrations of iron and iron-related proteins in lavage from both patients with idiopathic PAP and healthy control subjects.

## Methods

### Bronchoalveolar lavage in healthy subjects and patients with PAP

Alveolar lavage fluid was obtained from healthy volunteers by bronchoscopy with bronchoalveolar lavage (BAL) at the U.S. Environmental Protection Agency in Chapel Hill, NC under a protocol approved by the University of North Carolina [14]. Topical 2% lidocaine was used to anesthetize the upper airway and larynx. A fiberoptic bronchoscope was passed into the trachea, where 1% lidocaine was used for topical anesthesia. The lingula of the left lung was accessed, where the bronchoscope was wedged into a subsegment. BAL was performed with 250 mL of 0.9% saline injected serially in 50 mL aliquots. This procedure was repeated in the right middle lobe. Indices of iron homeostasis were measured in pooled samples of lavage fluid from the third, fourth, and fifth aliquots.

Lavage fluid was collected from patients with idiopathic PAP undergoing whole lung lavage for symptomatic treatment at the Duke University Medical Center [6,11]. The study protocol was approved by the Duke University Institutional Review Board. There was no clinical evidence of infection, including fever, chills, myalgias, elevated temperature, and leukocytosis, in any patient. Procedures were performed under general anesthesia using independent lung ventilation with a dual-lumen endotracheal tube. While the left lung was ventilated with 100% O_2_, the right lung was filled with warm 0.9% NaCl at 37°C. After drainage of 500 mL of lavage fluid, serial exchanges of 500 mL each were made using gravity augmented by chest physical therapy. Lavage was continued until the fluid was clear, which usually occurred after 15 liters. Samples used in this investigation were obtained from the second 1.5 L aliquot of collected fluid.

### Lavage iron, tranferrin, transferrin receptor, lactoferrin, ferritin, and protein

Since iron is taken up and released by all lung cells [15], iron homeostasis is maintained by an array of iron handling proteins, redox reactions, and storage mechanisms. Isolated parameters of iron content are not definitive; therefore a battery of measurements of uptake, storage and release proteins is needed to assess iron homeostasis. We measured lavage concentrations of iron, as well as levels of transferrin, transferrin receptor, lactoferrin, and ferritin as indices of the metal's homeostasis. In addition, iron and ferritin stains were performed in both lavage cells and lung tissue.

Iron concentration was measured with a standard colorimetric assay (Sigma, St. Louis, MO). Transferrin concentrations in lavage supernatants were analyzed using a commercially available kit, controls, and standards from INCSTAR Corporation (Stillwater, MN). TfR and ferritin were measured with commercial ELISA kits (R & D Systems, Minneapolis, MN) as were lactoferrin concentrations (Calbiochem, La Jolla, CA). Lavage protein was determined with Coomassie Plus Protein Assay Reagent (Pierce, Rockford, IL) using bovine serum albumin as the standard.

### Iron staining and ferritin immunohistochemistry

Lavage cells (at 1.0 × 10^6^/1.0 mL) were cytocentrifuged (0.2 mL) onto slides and stained for both iron and ferritin using Perl's Prussian Blue for iron (n = 4 for healthy subjects and 4 for PAP patients; all PAP cytospins were collected from non-smokers). Immunohistochemistry for ferritin was performed as reported with rabbit anti-human ferritin primary antibody (Dako, Carpinteria, CA) [16].

Human lung specimens were obtained from autopsy archives at Duke University Medical Center (fixed in 10% formalin). Immunohistochemistry was done on specimens from patients with primary PAP and specimens from patients with no lung disease listed as a diagnosis on the autopsy (n = 4 for no lung disease and 4 for PAP; all blocks were collected from non-smokers). Tissue sections were cut, floated on a protein-free water bath, mounted on silane treated slides (Fisher, Raleigh, NC), and air-dried overnight. Sections were stained for iron using Perl's Prussian Blue. Immunohistochemistry for ferritin was performed using an human anti-ferritin from Dako [17].

### Measurement of ascorbate, glutathione, and urate

Lavage samples were acidified by adding 35 μL of 60% perchloric acid to 1.0 mL of lavage and 1.5 mL of 4% percholoric acid to 0.5 mL of plasma. After 30 min centrifugation at 20,000 g, the supernatant was assayed for ascorbate and urate using high performance liquid chromatography (Waters RCM μ BondaPak C18 column, Millipore Corporation, Marlborough, MA) with electrochemical detection (BAS model LC-4B, Bioanalytical Systems, W. Lafayette, IN) [18]. Total glutathione was measured in the supernatant by quantifying nonprotein sulfhydryls, [19].

### Statistics

Data are expressed as mean values ± standard deviations. Differences between two groups were compared using T-tests of independent means. Significance was assumed at p < .05.

## Results

The healthy subjects that underwent a research bronchoscopy and bronchoalveolar lavage included 14 male and 6 female with a mean age of 26.5 +/- 3.3 years; all were non-smokers. In the group of patients with idiopathic PAP undergoing therapeutic whole lung lavage, there were 17 males and 3 females. The mean age in this cohort was 41.6 +/- 8.3 years; significantly older than the normal subjects. Among the PAP patients, there were 13 smokers/ex-smokers and 7 non-smokers.

The pooled BAL fluid from healthy volunteers was clear while that collected from patients with PAP was cloudy and ranged in color from beige to light pink. The amount of protein in the lavage collected from PAP patients was nearly ten-fold higher than that of the healthy volunteers (1,017 +/- 281 vs. 101 +/- 13 μgrams per mL).

**Figure 1:**
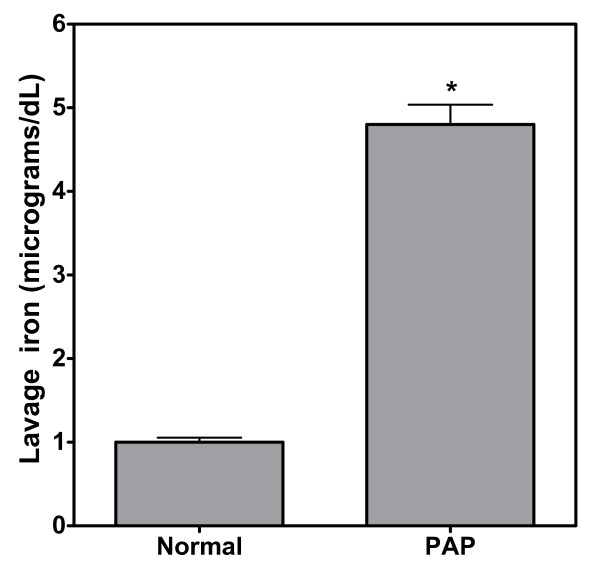
Levels of iron in bronchoalveolar lavage fluid (BAL) from normal volunteers and PAP patients. Metal concentrations in the bronchoalveolar fluid of PAP patients were elevated relative to values in healthy controls. All concentrations of iron were low relative to those normally found in blood (about 100 micrograms/dL). * significantly greater than normals; p < 0.05.

Iron is normally measurable in the BAL fluid of healthy subjects [20]. The iron concentrations in BAL from our healthy volunteers were similar to reported values and were about one hundredth of the serum value [20,21]. BAL iron concentrations from PAP patients were elevated five-fold relative to those of the healthy volunteers (Figure 1).

**Figure 2:**
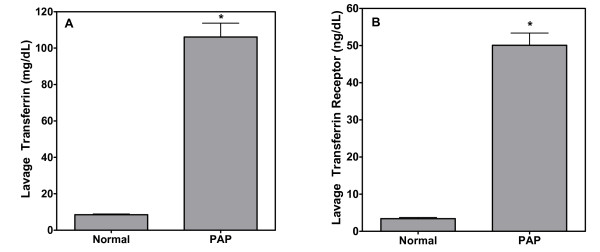
**A and B**. BAL transferrin and transferrin receptor concentrations in lavage from normals and PAP patients. Levels of this glycoprotein and its receptor were elevated in individuals with alveolar proteinosis. * significantly greater than normals.; p < 0.05.

Resident cells of the lower respiratory tract produce transferrin [22], a glycoprotein with anti-oxidant activity in the lung [23]. The transferrin receptor is also often found in measurable quantities in BAL fluid [21]. In this study, we found disparities between the patients and control subjects in BAL concentrations of both transferrin and transferrin receptor. The mean transferrin level in the lavage fluid of the PAP patients was at least ten-fold higher than that of the healthy volunteers (Figure 2A). Similarly, soluble transferrin receptor levels in the lavage of patients with PAP were also approximately ten-fold higher than in normal subjects (Figure 2B).

**Figure 3:**
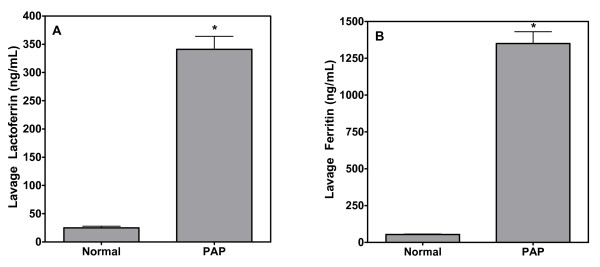
**A and B**. Concentrations of lactoferrin and ferritin in BAL fluid. Comparable to other iron-transport and storage proteins, lavage lactoferrin and ferritin were elevated in these patients. Ferritin concentrations in the PAP patients significantly exceeded normal values in serum of healthy subjects even with no correction for dilution occurring during the procedure. * significantly greater than normal, healthy subjects; p < 0.05.

Lactoferrin is a cationic metal-binding glycoprotein synthesized by myeloid cells and secretory epithelium commonly found in human mucosal secretions (eg. milk, tears, semen, and plasma) and in the specific granules of polymorphonuclear leukocytes. This glycoprotein can be an alternate route of iron transport, especially in inflammation [24]. Similar to transferrin, lactoferrin levels were elevated significantly in patients with PAP relative to healthy volunteers (Figure 3A).

We also measured ferritin, which stores iron in a non catalytically-reactive form. Iron sequestration by ferritin limits its capacity to generate free radicals and confers an antioxidant function to the protein [25,26]. Ferritin concentration in the normal lung is high, reflecting the direct interface of the organ with iron particulates in the external environment [17]. We found measurable concentrations of ferritin in BAL fluid from normal subjects, while ferritin levels in lavage fluid of PAP patients were unusually high (Figure 3B). Moreover, the ferritin levels measured in BAL fluid in PAP greatly exceed the normal serum levels of ferritin in humans (20 to 200 ng/mL).

In analyses of results from PAP patients only, there were no significant differences between smokers/ex-smokers, and non-smokers in lavage concentrations of iron, transferrin, transferrin receptor, lactoferrin, and ferritin.

**Figure 4:**
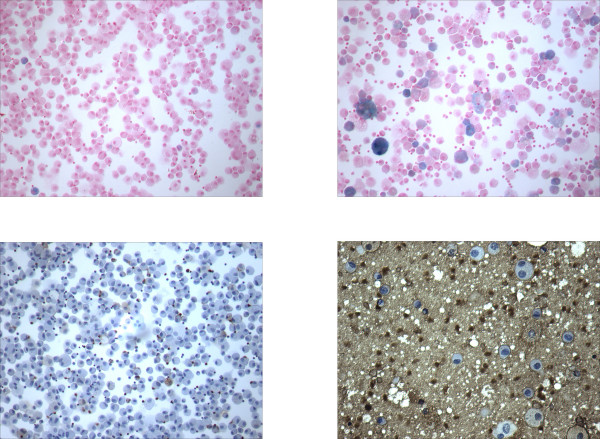
Stain of BAL cell pellets for iron (top) and ferritin (bottom) in healthy subjects (left) and patients with PAP (right). Sideromacrophages (stained blue) were rare in normals (top left) but increased in number among cells from PAP patients (top right). Similarly, those cells staining positively for ferritin (stained brown) were few in number in normals (bottom left) but were numerous in the cohort with PAP (bottom right). Extracellular ferritin appeared to be present. Magnification is approximately 100×.

BAL cell pellets from PAP patients showed significant iron accumulation relative to those from healthy subjects. Representative photomicrographs are shown in Figure 4. While only 1 to 2% of lavage cells from healthy subjects were found to be sideromacrophages, a large number of the cells in BAL fluid from PAP patients stained for iron. Similarly, macrophages collected from healthy subjects occasionally stained positively for ferritin (Figure 4), whereas ferritin staining was found in many lavage cells from PAP patients, and was also very intense in the extra-cellular debris.

**Figure 5:**
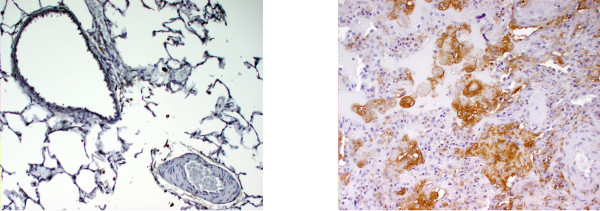
Blocks of archived lung from autopsies of patients dying without a lung disease showed normal staining for ferritin at the airways and among a few alveolar macrophages (left). Positive staining for ferritin was increased in lung tissue of patients diagnosed with PAP (right). Expression of the protein in the patients with PAP appears to include not only airway epithelium and macrophages but also material filling the alveolar spaces. Magnification approximates 200×.

Immunohistochemistry of lung sections also demonstrated significant quantities of ferritin (Figure 5). In lung sections from individuals dying without lung disease, ferritin was localized to the distal airway epithelium and alveolar macrophages. In the patients with PAP, ferritin expression was greatly increased in the distal lower respiratory tract, with intracellular staining of alveolar macrophages, and extracellular protein staining within the alveolar spaces.

**Figure 6:**
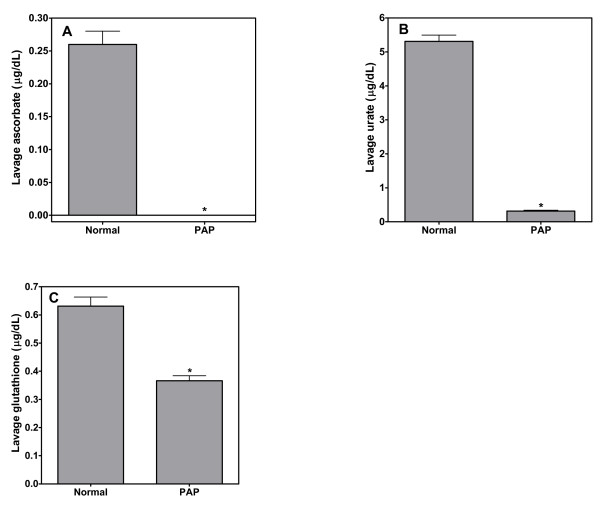
**A, B, and C**. Lavage concentrations of ascorbate, urate, and glutathione in healthy subjects and patients with PAP. There were decrements in the concentrations of all three anti-oxidants among patients with PAP. * significant decrease relative to normal, healthy subjects; p < 0.05.

Because the evidence of a disturbance in iron homeostasis was evident in the lungs of patient with PAP, we checked for evidence of oxidative stress by measuring the concentrations of ascorbate, urate, and glutathione in BAL fluid. The concentrations of all three anti-oxidants were significantly depressed in BAL fluid from PAP patients (Figures 6A,6B, and 6C). These results imply the presence of oxidative stress in the lower respiratory tract of PAP patients consistent with a presence of elevated concentrations of catalytically active iron.

## Discussion

This investigation describes a prominent disorder of iron homeostasis in the lower respiratory tract of individuals with primary PAP. Alterations in iron metabolism were evidenced by elevations in both concentrations of iron and levels of transferrin, transferrin receptor, lactoferrin, and ferritin in all patients with PAP. Histology and immunohistology also confirmed excess accumulation of iron and ferritin in the lungs of these patients. Corresponding to this iron accumulation, levels of ascorbate, urate, and glutathione were depressed consistent with the presence of oxidative stress. These changes in iron homeostasis and oxidative stress are not attributed to an increase in vascular permeability among patients with PAP; aberrant transport through a damaged blood-air barrier has been demonstrated not to occur in alveolar proteinosis [27].

Lung injury following exposures to both infectious agents and particles/fibers are iron-dependent [12,13]. Notably, surfactant accumulation in PAP is associated with certain infections and with inhalation of some particulates. This association suggested that iron homeostasis might be abnormal in PAP. A relationship between iron homeostasis, oxidative stress, and altered accumulation and function of surfactant has been suggested by prior investigations. For example, iron-loaded transferrin inhibits surfactant function in the airways of rabbits with acute respiratory failure, while apotransferrin has no effect on surfactant function [28]. In addition, iron-loaded transferrin incubated with H_2_O_2 _and a reducing agent leads to oxidative inactivation of surfactant, while transferrin without iron has the opposite, antioxidant effect [28]. Finally, administration of holotransferrin enhances acute respiratory failure in surfactant-depleted rabbits [28] while administration of apotransferrin is beneficial in the same animal model [28]. As the only difference between the exposures to holotransferrin and apotransferrin is the presence or absence of bound iron, it can be concluded that the metal presents an oxidative stress that leads to decrements in surfactant function and worsens respiratory failure.

Surfactant accumulation in rats after silica exposure also correlates with the concentration of iron complexed to the surface of the mineral oxide [29], supporting the association of metal-catalyzed oxidative stress with surfactant metabolism. Other mineral oxide dusts, including cristobalite, chrysotile, and kaolinite, have a comparable capacity to coordinate iron and present an oxidative stress to the lower respiratory tract. Similarly, these particles can be associated with elevated levels of extracellular surfactant [30]. Surfactant-enriched material appears to have a capacity to function as an *in vitro *target for oxidants catalyzed by Fe^3+ ^complexed to the surface of a particle [29].

Accumulation of surfactant in the lower respiratory tract occurs following exposure to other forms of oxidative stress which are also associated with an altered iron homeostasis. For example, environmental oxidants such as ozone and nitrogen dioxide increase the alveolar surfactant concentration [31,32]. In addition, chelates and metals other than iron can either redox cycle or disrupt iron metabolism, leading to oxidative stress and elevated surfactant levels in the lung [33-35]. Certain respiratory infections, such as tuberculosis and nocardiosis, are also causes of localized alveolar proteinosis, and our group has previously reported that a substantial fraction of patients with primary PAP have positive BAL cultures for *Mycobacterium avium complex (11)*.

An alteration of iron homeostasis and oxidative stress are found in the lung during certain infections and particle/fiber exposures, and for the first time we report idiopathic PAP among the lung disorders with disturbed iron handling. That both oxidative stress and altered iron homeostasis are present in the lungs of patients with PAP is associative; however, a causal link is strongly suggested by the fact that PAP is closely associated with stimuli that create significant oxidative stress in the lower respiratory tract. Oxidative injury following the exposure of the lung to microorganisms or particulates could contribute to disease pathogenesis in primary PAP by causing surfactant oxidation and dysfunction, and its subsequent accumulation [29].

An accumulation of iron in lung tissue with aging has been demonstrated [36]. Some increase in lavage iron is certainly predicted with aging but this has not been confirmed in any study. Based on these reported changes in tissue non-heme iron concentrations, the differences in age observed in this study between the healthy subjects and PAP patients cannot account for the observed disparities in iron and iron-related proteins. Relative to aging, smoking will have a greater impact on lavage concentrations of iron and iron-related proteins. Iron concentrations also increase in the lavage from cigarette smokers [37]. Concentrations of specific iron-related proteins in lavage fluid have not been reported except for ferritin and this storage protein increased in the lavage fluid of smokers [37,38]. Smoking may contribute to some portion of the differences in iron and iron-related proteins observed in this study between the healthy subjects and PAP patients. However, smoking fails to explain disparities between these two cohorts as there were no significant differences in lavage concentrations of iron and iron-related proteins between smokers/ex-smokers and non-smokers among the PAP patients. In addition, the magnitude of changes observed reported in previous studies among smokers does not approach those observed in this investigation among PAP patients.

Lavage iron and ferritin concentrations are similarly elevated among patients with acute respiratory distress syndrome [39] and pneumoconiosis[40], and following transplantation [41]. However, the disturbance in iron homeostasis among PAP patients reported in this investigation is exceptional in its magnitude with 5- to 20-fold elevations in concentrations of the metal and specific iron-related proteins.

Since reactive oxygen species (ROS) generated by excess iron damages biological molecules, optimal homeostasis requires extremely low concentrations of free iron. The transport and storage of iron is with all coordination sites complexed to prevent excess ROS generation. The host responds to elevated concentrations of available iron by sequestering it in the storage protein ferritin. This requires transporting the metal intracellularly, usually by the glycoprotein transferrin, which uses a membrane-bound receptor TfR. Lactoferrin-mediated metal transport is an alternative mechanism for metal transfer after exposures to microbes and particles/fibers, and is important for transport of iron to ferritin in monocytes and macrophages during inflammation. A novel aspect of our data is the finding that TfR in PAP lavage fluid is increased despite elevated soluble iron levels in the lung. With exposure to iron, many cell types rapidly down regulate TfR expression to limit iron uptake. However, with elevations in available iron observed during inflammation, monocytes and macrophages actually increase TfR expression [42,43]. Increased TfR in the BAL of PAP patients may reflect a defensive attempt to transport and sequester elevated concentrations of metal that occur in inflammatory injuries.

## Conclusion

We conclude that primary PAP is associated with an altered iron homeostasis in the lung. Our findings suggest the presence of metal-catalyzed oxidative stress in this rare lung disease. They also imply that, rather than being a uniform disease with a single etiology, PAP can be a pattern of injury associated with loss of iron homeostasis in the lower respiratory tract and a resultant oxidative stress.

## Competing interests

The author(s) declare that they have no competing interests.

## Authors' contributions

AJG contributed to conception and design, acquisition of data, and analysis and interpretation of data. He was involved in drafting the manuscript and has given final approval of the version submitted.

JS contributed to acquisition of data and analysis and interpretation of data. She was involved in drafting the manuscript and has given final approval of the version submitted.

KC contributed to acquisition of data and analysis and interpretation of data. She was involved in drafting the manuscript and has given final approval of the version submitted.

JR contributed to acquisition of data and analysis and interpretation of data. She was involved in drafting the manuscript and has given final approval of the version submitted.

VLR contributed to acquisition of data, and analysis and interpretation of data. He was involved in drafting the manuscript and has given final approval of the version submitted.

CAP contributed to conception and design, acquisition of data, and analysis and interpretation of data. He was involved in drafting the manuscript and has given final approval of the version submitted.

MSC contributed to conception and design, acquisition of data, and analysis and interpretation of data. She was involved in drafting the manuscript and has given final approval of the version submitted.
